# Mek activity is required for ErbB2 expression in breast cancer cells detached from the extracellular matrix

**DOI:** 10.18632/oncotarget.22194

**Published:** 2017-10-31

**Authors:** Iman A. Khan, Byong H. Yoo, Janusz Rak, Kirill V. Rosen

**Affiliations:** ^1^ Department of Biochemistry and Molecular Biology, Dalhousie University, Halifax, Canada; ^2^ Department of Pediatrics, Dalhousie University, Halifax, Canada; ^3^ Department of Pediatrics, McGill University, Montreal, Canada; ^4^ The Research Institute of the McGill University Health Centre, Montreal Children's Hospital, Montreal, Canada

**Keywords:** extracellular matrix, three-dimensional tumor growth, ErbB2, Mek, trastuzumab

## Abstract

Detachment of non-malignant epithelial cells from the extracellullar matrix (ECM) triggers their growth arrest and apoptosis. Conversely, carcinoma cells can grow without adhesion to the ECM. This capacity for anchorage-independent growth is thought to be critical for tumor progression. ErbB2/Her2 oncoprotein is overproduced by a significant fraction of breast cancers and promotes anchorage-independent tumor cell growth by poorly understood mechanisms. In an effort to understand them we found that in order to produce ErbB2, detached breast cancer cells require the activity of an ErbB2 effector protein kinase Mek and that Mek-driven ErbB2 expression is neccesary for anchorage-independent growth of such cells. We observed that Mek inhibition does not alter ErbB2 mRNA levels in detached cancer cells and that ErbB2 protein loss induced by this inhibition can be blocked by a lysosomal inhibitor. We also noticed that an increase of the density of cancer cells detached from the ECM downregulates a Mek effector protein kinase Erk and causes ErbB2 loss. Those cells that survive after ErbB2 loss display resistance to trastuzumab, an anti-ErbB2 antibody used for ErbB2-positive breast cancer treatment. Thus, Mek-induced ErbB2 stabilization in detached breast cancer cells is critical for their ability to grow anchorage-independently and their trastuzumab sensitivity.

## INTRODUCTION

15-20% of breast tumors overproduce ErbB2/Her2 oncoprotein which drives the progression of these malignancies [1]. In most cases ErbB2 overproduction occurs due to the amplification of the ErbB2 gene [2]. ErbB2, a member of the ErbB family of receptor tyrosine kinases, triggers numerous oncogenic signals in cancer cells by binding other members of the family, such as Epidermal Growth Factor Receptor (EGFR) or ErbB3 [2]. The indicated heterodimers trigger various signalling events in the cells, e.g. activation of a protein kinase Raf which in turn induces a protein kinase Mek [2]. Mek then phosphorylates and thus activates a protein kinase Erk [3] which phosphorylates diverse cellular proteins and thereby controls their activity.

One critical feature of primary and disseminated breast tumors, including those overproducing ErbB2, is their ability to grow in a three-dimensional manner [4]. Such growth requires the ability of cancer cells to survive without adhesion to the extracellular matrix (ECM) [5]. This notion is based on the fact that normal basal and luminal breast epithelial cells are attached to the ECM in the breast [6, 7]. Detachment causes their growth arrest [8] and death by apoptosis [9]. The latter phenomenon is called anoikis [10]. In contrast, breast tumors grow, invade adjacent tissue and metastasize to other organs as three-dimensional multicellular masses in which the cells are not properly attached to the ECM but remain viable and capable of proliferation [9]. Numerous lines of evidence indicate that the ability oftumor cells, including breast cancer cells, to survive and grow without adhesion to the ECM is critical for tumor progression [9]. First, cancer cells survive and grow without attachment to the ECM as colonies in soft agar. This ability represents a “gold standard” for malignant transformation [11, 12]. Second, major oncoproteins, e.g. ErbB2 [4], Ras [13, 14] and β-catenin [15], promote anchorage-independent survival and growth of cancer cells. Moreover, approaches suppressing anchorage-independent growth of malignant cells block their ability to form primary tumors [16–20] and metastases [16, 19, 21–23]. Finally, spontaneous acquisition of the ability to grow in a three-dimensional manner is sufficient for the attainment of *in vivo* tumorigenicity by cancer cells [24]. Also importantly, formation of three-dimensional multicellular masses was found to render cancer cells resistant to chemotherapeutic agents [25]. This phenomenon is called “multicellular drug resistance” [25].

Mechanisms by which ErbB2 promotes three-dimensional growth of breast cancer cells are understood in part. One such mechanism has emerged from our work [26]. We found that ErbB2 blocks anoikis of breast cancer cells by downregulationg a protein Perp that triggers apoptosis by an unknown mechanism. Of note, it is known that detachment of non-malignant breast epithelial cells triggers lysosmal degradation of an ErbB2 signalling partner EGFR and that ErbB2-induced Mek activation prevents this degradation in detached breast cancer cells [27]. We observed that the effect of ErbB2/Mek on EGFR is required for ErbB2-induced Perp downregulation in the indicated cells [26].

In an effort to further understand the mechanisms that control ErbB2-dependent three-dimensional growth of breast cancer cells we found in this study that Mek activity is required for the expression of ErbB2 itself in ErbB2-positive breast cancer cells detached from the ECM. We observed that in the absence of Mek activity ErbB2 undergoes lysosomal degradation in detached cells. We also demonstrate here that Mek-induced ErbB2 upregulation is required for anchorage-independent growth of malignant breast epithelial cells. Finally, we show that as the number of detached breast tumor cells composing a three-dimensionally growing cellular mass increases, Mek activity and ErbB2 expression are lost and the resulting ErbB2-deficient cells display resistance to trastuzumab, an anti-ErbB2 antibody normally used for treatment of ErbB2-positive breast cancer. Thus, Mek-dependent ErbB2 expression in detached breast cancer cells is critical for their ability to grow without adhesion to the ECM and for their trastuzumab sensitivity.

## RESULTS

### Mek activity is required for ErbB2 expression in detached breast cancer cells

**Figure 1:**
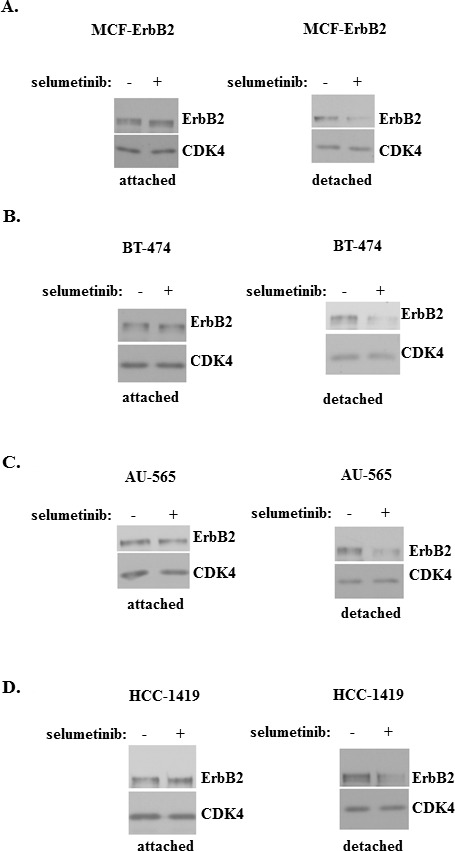
Mek activity is required for ErbB2 expression in breast cancer cells detached from the ECM MCF-ErbB2 **(A)**, BT-474 **(B)**, AU-565 **(C)** and HCC-1419 cells **(D)** were cultured attached to (attached) or detached from (detached) the ECM in the presence of DMSO (−) or 1μM selumetinib (+) for 5h and assayed for ErbB2 expression by western blot. CDK4 was used as a loading control.

One model that we used to study the role of Mek in the ability of ErbB2-expressing breast cancer cells to grow without adhesion to the ECM represents MCF-ErbB2 cells derived from non-malignant breast epithelial cells MCF10A by infection with a wild type ErbB2-encoding retrovirus [26, 28]. Unlike the parental MCF10A cells which undergo anoikis after detachment and do not form colonies in soft agar, MCF-ErbB2 cells are anoikis-resistant and efficiently grow in soft agar [26]. We found that treatment of MCF10A cells with a widely used highly specific Mek inhibitor selumetinib [29, 30] strongly downregulates ErbB2 in detached MCF-ErbB2 cells but has no impact on ErbB2 in the attached cells (Figure 1A). The effect of selumetinib on ErbB2 was not unique to MCF-ErbB2 cells as we found that the Mek inhibitor downregulates ErbB2 in detached ErbB2-positive human breast cancer cell lines BT-474, AU-565 and HCC-1419 [31, 32] but has no effect on ErbB2 levels when these cells are attached to the ECM (Figure 1B-1D). (Changes in the ErbB2 protein levels observed in Figure 1A-1D are quantified in Supplementary Figure 1). Thus, Mek activity is required for ErbB2 expression in breast cancer cells detached from the ECM.

### Mek-dependent ErbB2 expression is required for anchorage-independent growth of malignant breast epithelial cells

**Figure 2:**
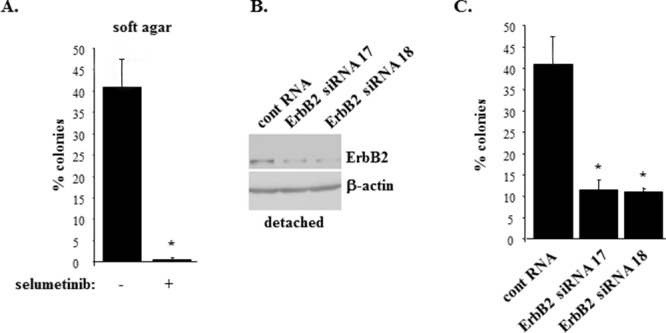
Inhibition of Mek or ErbB2 knockdown block anchorage-independent growth of ErbB2-overproducing breast epithelial cells **(A)**. MCF-ErbB2 cells were plated in monolayer culture or in soft agar in the presence of DMSO (−) or 1μM selumetinib (+) and allowed to form colonies which were counted 10 days later. % colonies was calculated as a ratio between the number of colonies in soft agar to that in monolayer x100%. **(B)**. MCF-ErbB2 cells were transfected with 25nM control RNA or 25 nM ErbB2-specific siRNA (ErbB siRNA) 17 or 18, cultured detached from the ECM for 24 and assayed for ErbB2 expression by western blot. β-actin was used as a loading control. **(C)**. Cells treated as in (B) were assayed as in (A). The data in (A) and (C) represent the average of the triplicates plus SD. ^*^ indicates that p value was < 0.05.

We further tested whether selumetinib blocks the ability of ErbB2-overproducing breast epithelial cells MCF-ErbB2 to grow without adhesion to the ECM. Of note, Mek inhibition is known to trigger various feedback anti-apoptotic mechanisms in cancer cells (e.g. ErbB3 upregulation [33]) which could in principle promote their survival. Thus, the effect of Mek inhibition on anchorage-independent growth of ErbB2-positive cells cannot be predicted. We observed that selumetinib-induced ErbB2 downregulation in MCF-ErbB2 cells (see Figure 1A) is accompanied by loss of the ability of these cells to grow anchorage-independently as colonies in soft agar (Figure 2A). Moreover, we found that ErbB2 knockdown in MCF-ErbB2 cells by small interfering (si) RNAs can mimic the effect of selumetinib on these cells, i.e. such knockdown strongly blocks their soft agar growth (Figure 2B, 2C). (Changes in the ErbB2 protein levels observed in Figure 2B are quantified in Supplementary Figure 2).

**Figure 3:**
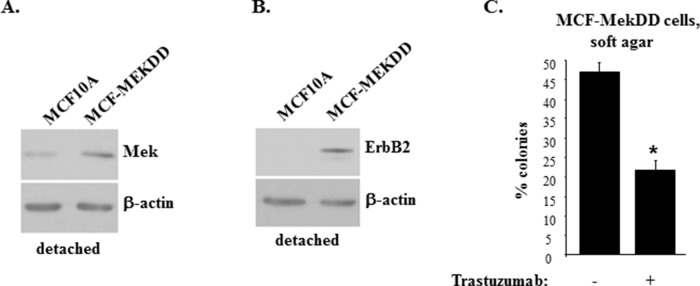
Mek-induced ErbB2 uregulation promotes anchorage-independent growth of breast epithelial cells Indicated cells were cultured detached from the ECM for 3h and assayed for Mek **(A)** or ErbB2 **(B)** expression by western blot. β-actin was used as a loading control. **(C)**. MCF-MekDD cells were plated in monolayer culture or in soft agar in the absence (−) or in the presence (+) or 5μg/ml trastuzumab and allowed to form colonies which were counted 10 days later. % colonies was calculated as a ratio between the number of colonies in soft agar to that in monolayer x100%. The data represent the average of the triplicates plus SD. ^*^ indicates that p value was < 0.05.

We further tested the effect of Mek on ErbB2 in detached MCF10A cells and a variant of these cells MCF-MekDD generated by infection of the former cells with the retrovirus encoding an activated Mek mutant [26, 28]. We found that as expected MCF-MekDD cells displayed higher total Mek levels than the parental MCF10A cells (Figure 3A) and showed much higher ErbB2 levels than MCF10A cells when these cell lines were detached from the ECM (Figure 3B). (Changes in protein levels observed in Figure 3A, 3B are quantified in Supplementary Figure 3). We further observed that MCF-MekDD cells are capable of growing anchorage-independently in soft agar and that treatment with trastuzumab, a therapeutic anti-ErbB2 antibody [34], significantly reduced the ability of the indicated cells to grow in this manner (Figure 3C). Thus, one function of Mek in detached breast cancer cells is to support ErbB2 expression. In the absence of active ErbB2 the ability of Mek to promote anchorage-independent growth of such cells is significantly reduced.

### Mek does not upregulate ErbB2 mRNA in detached breast cancer cells

**Figure 4:**
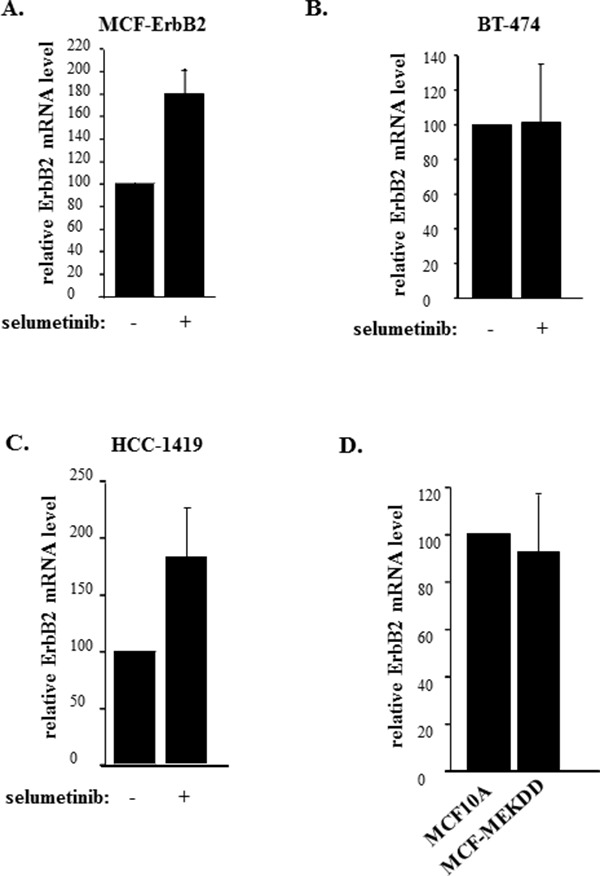
Mek does not control ErbB2 mRNA expression in breast cancer cells detached from the ECM MCF-ErbB2 **(A)**, BT-474 **(B)** and HCC-1419 **(C)** were cultured detached from the ECM (detached) in the presence of DMSO (−) or 1μM selumetinib (+) for 5h and assayed for ErbB2 mRNA levels by qPCR. **(D)**. MCF10A and MCF-MekDD cells were cultured detached from the ECM for 3h and assayed for ErbB2 mRNA levels by qPCR. ErbB2 mRNA levels observed in (A-D) were normalized by the levels of 18S rRNA which were also determined by qPCR. The resulting levels of the ErbB2 mRNA in the DMSO-treated cells (A-C) or MCF10A cells (D) were designated as 100%. The data in (A, C) represent the average of three and those in (B, D) the average of four independent experiments plus SE.

In an effort to understand the mechanisms by which Mek controls ErbB2 expression in detached breast cancer cells we found that selumetinib does not downregulate ErbB2 mRNA in detached MCF-ErbB2 cells. To the contrary, the Mek inhibitor upregulated this mRNA in the indicated cells (Figure 4A). We also observed that selumetinib has no effect on the ErbB2 mRNA in detached human breast cancer cells BT474 (Figure 4B). Moreover, the Mek inhibitor upregulated, rather than downregulated, the ErbB2 mRNA in detached human breast cancer cells HCC-1419 (Figure 4C). Finally, an activated Mek mutant, which upregulates ErbB2 protein in detached MCF-10A cells (Figure 3B), did not upregulate the ErbB2 mRNA in these cells (Figure 4D). Hence, since selumetinib downregulates ErbB2 protein in detached breast cancer cells (Figure 1), whereas activated Mek upregulates ErbB2 protein in detached breast epithelial cells (Figure 3B), the effect of Mek on ErbB2 protein in such cells does not involve changes in the ErbB2 mRNA.

### ErbB2 loss following Mek inactivation in detached breast cancer cells can be blocked by a lysosomal inhibitor

**Figure 5:**
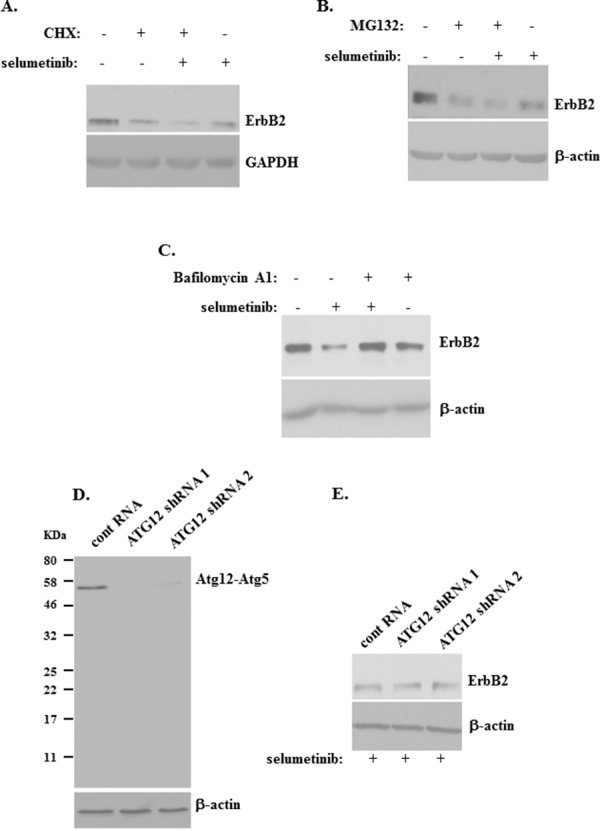
ErbB2 downregulation induced by a Mek inhibitor in detached breast cancer cells can be blocked by a lysosomal inhibitor **(A)** BT-474 cells were cultured detached from the ECM in the presence of DMSO (−) or 1μM selumetinib (+) or 10μg/ml cyclohexamide (CHX) (+) for 3h and assayed for ErbB2 expression by western blot. **(B)** BT-474 cells were cultured detached from the ECM in the presence of DMSO (−) or 1μM selumetinib (+) or 10μg/ml MG132 (MG132) (+) for 5h and assayed for ErbB2 expression by western blot. **(C)** BT-474 cells were cultured detached from the ECM in the presence of DMSO (−) or 1μM selumetinib (+) or 100 nM Bafilomycin A1 (+) for 5h and assayed for ErbB2 expression by western blot. **(D)** BT-474 cells were infected with lentiviruses encoding the control RNA (cont RNA) or Atg12-specific shRNA 1 or 2 (Atg12 shRNA 1 or 2) and a puromycin resistance gene, the cells were expanded in the presence of puromycin, and the resulting stable cell lines were assayed for Atg12 expression by western blot by use of the anti-Atg12 antibody. Position of the Atg12-Atg5 covalent complex (Atg12-Atg5) on the gel and that of the molecular weight markers (KDa) is indicated. **(E)** Cells generated as in (D) were cultured detached from the ECM in the presence of 1μM selumetinib (+) for 5h and assayed for ErbB2 levels by western blot. GAPDH (A) and β-actin (B-E) was used as a loading control.

Given that Mek inhibition downregulates ErbB2 protein in detached breast cancer cells without downregulating the ErbB2 mRNA, we tested whether Mek inactivation blocks ErbB2 protein stability in these cells. To this end, we treated detached human breast cancer cells BT474 with a protein synthesis inhibitor cyclohexamide [35, 36]. As could have been expected in the absence of *de novo* protein synthesis, ErbB2 levels were reduced to some extent by this treatment (Figure 5A). Moreover, cyclohexamide noticeably enhanced selumetinib-induced ErbB2 protein loss in detached breast cancer cells (Figure 5A). These data are consistent with a scenario that the absence of *de novo* protein synthesis accelerates selumetinib-induced ErbB2 protein loss resulting from selumetinib-induced ErbB2 protein degradation.

We further tested whether Mek inhibition promotes covalent binding of ErbB2 to a protein ubiquitin and subsequent ErbB2 proteasomal degradation (a known mechanism of protein turnover [37]) in detached breast cancer cells. To this end, we treated detached BT474 cells with selumetinib in the absence and in the presence of a proteasomal inhibitor MG132 [37]. We observed that treatment with MG132 for 2h (not shown) or 5h (Figure 5B) does not block the effect of selumetinib on ErbB2 in these cells. Hence, ErbB2 ubiquitination and subsequent proteasomal degradation does not seem to be involved in the effect of selumetinib on ErbB2 in detached breast cancer cells.

It is known that Mek inhibition accelerates lysosomal degradation of EGFR [27], an ErbB2 signalling partner [2]. Moreover, lysosomal degradation is an established mechanism of ErbB2 protein turnover in certain circumstances. For example, it was found that a therapeutic HSP90 inhibitor [38] or an inhibitor of phosphatidylcholine-specific phospholipase C [39] trigger lysosomal ErbB2 degradation in breast cancer cells. To test whether Mek inhibition promotes degradation of ErbB2 in the lysosomes of detached breast cancer cells we treated detached BT474 cells with selumetinib in the absence and in the presence of a lysosomal inhibitor Bafilomycin A1 [40, 41]. We observed that Bafilomycin A1 strongly blocks selumetinib-induced ErbB2 downregulation in these cells (Figure 5C). Collectively, our data are consistent with a scenario that Mek activity prevents lysosomal ErbB2 degradation in detached breast cancer cells.

Of note, it was proposed that in certain circumstances ErbB2 can covalently bind ubiquitin, and that such ubiquitination further triggers proteasome-mediated cleavage of the intracellular ErbB2 region [42]. This, in turn was suggested to induce internalization and subsequent sorting of ErbB2 to the lysosomes [42]. Our data showing that selumetinib-induced ErbB2 degradation is proteasome-independent (Figure 5B) indicate that the mechanism outlined above is unlikely involved in the effect of selumetinib on ErbB2 in breast cancer cells detached from the ECM.

Another mechanism of protein delivery to the lysosomes involves protein engulfment by vacuole-like structures called autophagosomes which can fuse with the lysosomes and thus deliver its content into the latter organelles where the indicated content is degraded. This process is called autophagy [43]. To test whether autophagosomes mediate the effect of Mek inhibition of ErbB2 in detached BT474 cells we knocked down a critical mediator of autophagosome formation Atg12 [43] in these cells by infecting them with lentiviruses encoding two different Atg12-specific small hairpin (sh) RNAs (Figure 5D). Atg12 mediates autophagosome formation by covalently binding another critical autophagy mediator Atg5 [43], and the majority of cellular Atg12 is often constitutively conjugated with Atg5 even in the absence of autophagy [37]. Similar to what was observed by others [37], we found that essentially all Atg12 (whose molecular weight is approximately 20KD) is present in BT474 cells as species with a molecular weight of approximately 55KD (Figure 5D) which is well known to represent the Atg12-Atg5 covalent complex [37, 43]. Introduction of Atg12-specific shRNAs in the cells resulted in a strong downregulation of the indicated complex (Figure 5D) but did not upregulate ErbB2 in the cells when they were treated with selumetinib in the absence of adhesion to the ECM for 2h (not shown) or 5h (Figure 5E). These data are consistent with a scenario that autophagy is not involved in the effect of Mek inhibition on ErbB2. (Changes in protein levels observed in Figure 5 are quantified in Supplementary Figure 4). In summary, our data indicate that Mek blocks lysosomal ErbB2 degradation in detached breast tumor cells.

### The activity of the Mek/Erk signalling pathway, ErbB2 expression and trastuzumab sensitivity can be blocked by an increased density of detached breast tumor cells

Our data indicate that Mek inhibition blocks ErbB2 expression in detached breast cancer cells. In search for physiologically relevant circumstances under which this scenario could take place we reasoned that ErbB2 loss represents an established mechanism of breast cancer resistance to treatment with a therapeutic anti-ErbB2 antibody trastuzumab [44, 45]. Moreover, it was found that breast tumor cells circulating in patient's blood are ErbB2-positive in a significant number of cases whereas the cells composing respective primary tumors are often ErbB2-negative [46]. Finally, it was found that growth of tumors formed by ErbB2-positive breast cancer cells injected in immunodeficient mice is blocked by trastuzumab significantly more efficiently if the drug is administered immediately after cell injection (before the tumor mass is formed) compared to the situation when the drug is administered after a tumor mass is established [47]. Thus, we speculated that increase in the cancer cell density within a three-dimensional tumor mass can block the activity of Mek or that of its substrates and cause inhibition of ErbB2 expression. This in turn could render breast cancer cells trastuzumab-resistant.

**Figure 6:**
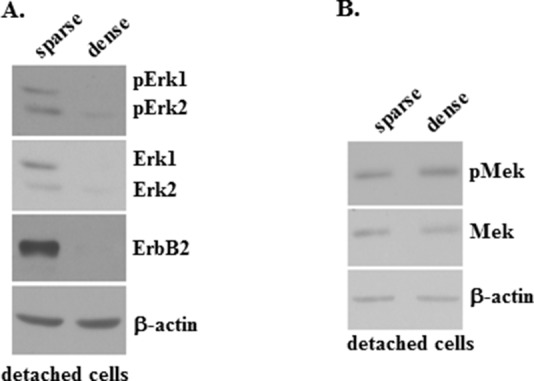
pErk, Erk and ErbB2 protein expression is blocked by an increased density of detached breast tumor cells **(A, B)** BT-474 cells were cultured detached from the ECM for 6 days at a concentration 12500 cells/ml (sparse) or 125000 cells/ml (dense) and assayed for phospho-Erk1 (pErk1), phospho-Erk2 (pErk2), Erk1, Erk2 and ErbB2 protein expression (A) or phospho-Mek (pMek) and Mek protein expression (B) by western blot. β-actin was used as a loading control in (A, B).

To test whether an increased density of detached breast cancer cells can block the activity of Mek or that of its substrate Erk and inhibit ErbB2 expression we compared the levels of Erk1 and Erk2 (the two Mek substrates [3]), phospho-Erk1 and phospho-Erk2 as well as that of ErbB2 in human breast cancer cells BT-474 detached from the ECM grown as a “sparse” culture to those in detached BT-474 cells grown at a 10 times higher concentration (which we refer to as a “dense” culture). The cells in the sparse culture formed multiple relatively small size spheroids whereas those cultured in the dense culture represented one large multicellular aggregate (not shown). We found that densely grown cells display much lower levels of Erk1 and Erk2 proteins as well as those of phospho-Erk1 and phospho-Erk2 than the sparsely grown cells (Figure 6A). Of note, the increase in cell density did not significantly reduce the levels of the Erk1 and Erk2 mRNAs (Supplementary Figure 5) indicating that the observed downregulation of Erk1 and Erk2 occurred at the protein, rather than at the mRNA level. Unlike the case with Erk and phospho-Erk, the indicated increase in the cell density did not significantly alter the cellular protein levels of Mek and phospho-Mek (Figure 6B). We further found that an increase in the density of detached cells resulted in a dramatic reduction of ErbB2 protein levels (Figure 6A). (Changes in the levels of proteins observed in Figure 6 are quantified in Supplementary Figure 6).

**Figure 7:**
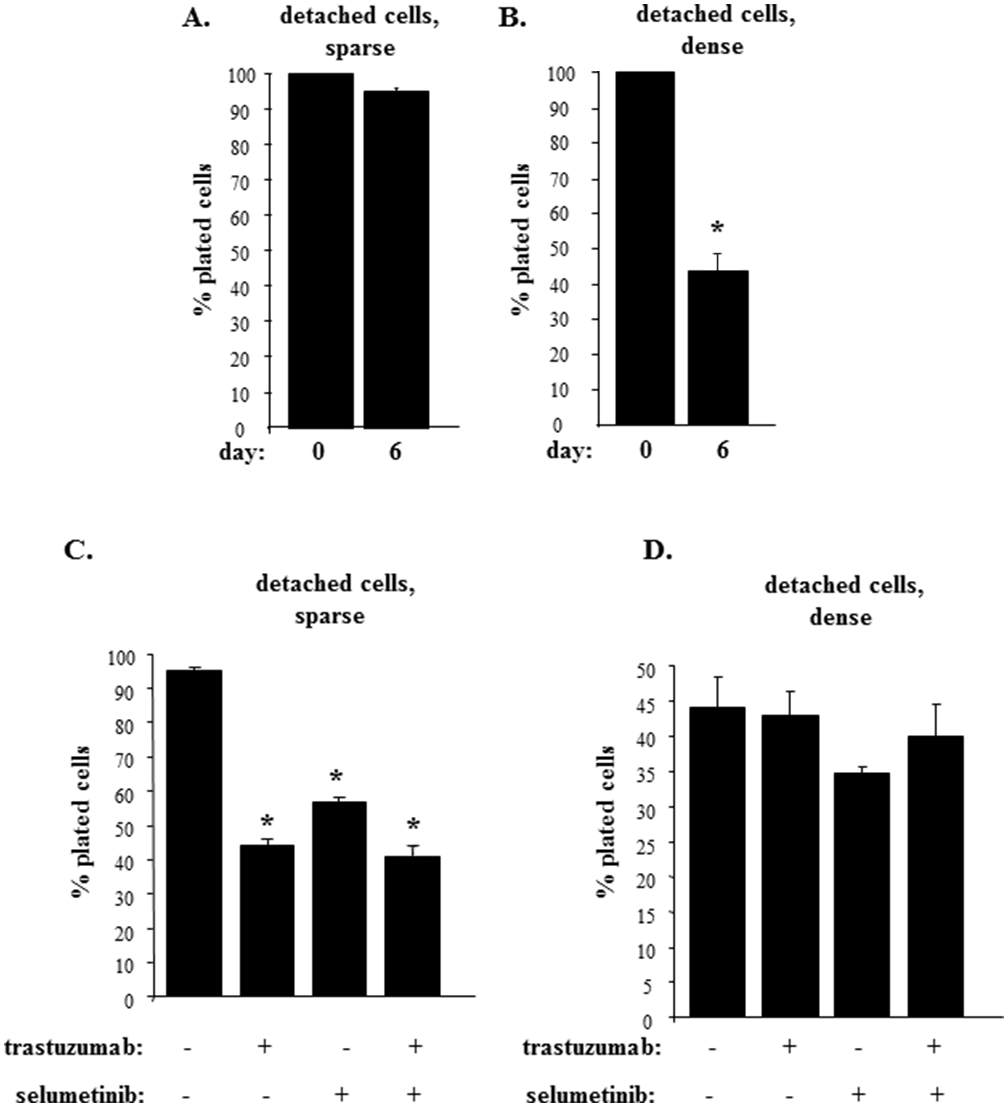
Trastuzumab sensitivity is blocked by an increased density of detached breast tumor cells **(A, B)**. BT-474 cells were cultured detached from the ECM for 6 days at a concentration 12500 cells/ml (sparse) (A) or 125000 cells/ml (dense) (B) for 6 days and the cells were counted. **(C, D)**. BT-474 cells were cultured detached from the ECM for 6 days at a concentration 12500 cells/ml (sparse) (C) or 125000 cells/ml (dense) (D) for 6 days in the presence of DMSO (−) or 1μM selumetinib (+) or 5μg/ml trastuzumab (+) and the cells were counted. % plated cells was calculated as that of the cells plated at day 0. The data represent the average of the triplicates (A, B, D) or that of the duplicates (C) plus SD. ^*^ indicates that p value was < 0.05.

We subsequently noticed that culturing detached cells sparsely did not reduce their number compared to the number of those initially plated (Figure 7A). In contrast, culturing the cells at high density significantly reduced their number compared to the number of initially plated cells (Figure 7B). These data are consistent with our observations shown in Figures 1–3 indicating that ErbB2 is requited for anchorage-independent growth of these cells. Of note, even though plating the cells in the dense culture noticeably reduced their number, a substantial fraction of the cells remained viable (Figure 7B). One possible explanation of the fact that these cells survived in the absence of ErbB2 is that in addition to ErbB2 gene amplification [32], other oncogenic changes present in the cells contribute to their ability to grow anchorage-independently. For example, BT474 cells also carry a loss-of-function mutation of the p53 tumor suppressor gene [48], and loss of p53, a well known inhibitor of apoptosis and cell proliferation [49], could contribute to the ability of detached cells that did not succumb to ErbB2 loss (Figure 6A, 7B) to survive and grow in the dense cell culture.

We further observed that the number of cells grown in the sparse culture (and producing relatively high ErbB2 amounts) was significantly reduced by trastuzumab treatment (Figure 7C). We also found that treatment with selumetinib (which downregulates ErbB2 in these sells (Figure 1B) reduced the number of detached cells to a degree similar to that observed in case of trastuzumab treatment (Figure 7C). These data are consistent with a notion that has emerged from our studies (Figure 1–3) that Mek inhibition blocks anchorage-independent growth of breast cancer cells at least in part by downregulating ErbB2. We further noticed that a combination of selumetinib and trastuzumab had the same impact on the growth of detached cells as each drug alone (Figure 7C). The latter data support a scenario that once selumetinib blocks ErbB2 expression in the cells, trastuzumab (that no longer has a target) does not affect growth of those cells that are still present in the culture after selumetinib treatment.

Remarkably, trastuzumab treatment failed to reduce the number of ErbB2-deficient cells that were present in the dense culture (Figure 7D). In addition, selumetinib or its combination with trastuzumab did not reduce the number of detached densely grown cells (Figure 7D). Thus, increased density of detached breast cancer cells strongly reduces Erk, phospho-Erk and ErbB2 levels in these cells and renders those cells that remain viable trastuzumab-resistant.

In summary, we have identified a novel mechanism of the regulation of ErbB2 expression in breast cancer cells. When the cells detach from the ECM this expression becomes strongly dependent on Mek activity. Furthermore, the ability of Mek to promote anchorage-independent growth of such cells requires Mek-induced ErbB2 upregulation. Once Mek activity is blocked in breast cancer cells, the cells lose ErbB2 and those cells that survive become trastuzumab-resistant.

## DISCUSSION

We have identified in this study a novel mechanism by which a protein kinase Mek controls three-dimensional growth of ErbB2-positive breast cancer cells. Mek promotes growth of these cells without adhesion to the ECM by supporting ErbB2 expression. This function of Mek is somewhat unexpected. It is well known that the indicated protein kinase can promote Erk-dependent phosphorylation and thus alter the activity of numerous transcription factors [50]. One easily conceivable scenario for the role of Mek in anchorage-independent growth of tumor cells is that once activated downstream of ErbB2, Mek alters the activity of those transcription factors that directly control the expression of the regulators of cell growth and survival and thus promotes growth of these cells without adhesion to the ECM. However, this, at least in part, appears not to be the case. We found that the ability of breast epithelial cells producing an activated Mek mutant to grow anchorage-independently is substantially impaired if the activity of ErbB2 (which is strongly upregulated by Mek in these cells) is blocked by trastuzumab (Figure 3). Hence three-dimensional growth of these cells significantly relies on the ability of Mek to upregulate ErbB2, rather than (or in addition to) the ability of Mek to promote phosphorylation of transcription factors that directly control levels of proteins regulating cells growth and survival. We and others found that ErbB2 blocks anoikis of breast cancer cells by downregulating pro-apoptotic proteins Perp [26] and Bim [51] in a Mek-dependent manner. It would be of significant interest to test whether the effect of Mek on these proteins requires Mek-driven ErbB2 upregulation in the indicated cells.

Our data are consistent with a scenario that Mek promotes ErbB2 expression in breast cancer cells by blocking lysosomal ErbB2 degradation. These data are supported by findings that ErbB2 can be degraded by the lysosomes in breast cancer cells in response to various stimuli. For example, it was established that a therapeutic HSP90 antagonist can trigger lysosomal ErbB2 degradation in breast cancer cells [38]. Moreover, an inhibitor of phosphatidylcholine-specific phospholipase C can promote lysosomal ErbB2 degradation in these cells as well [39]. A similar mode of regulation was identified for an ErbB2 signalling partner EGFR. It was found that detachment of non-malignant breast cancer cells promotes lysosomal EGFR degradation [27]. Perhaps not by coincidence, it was observed that ErbB2 blocks lysosomal EGFR degradation by activating Mek [27].

What is the potential physiological relevance of our findings? It is known that ErbB2 levels are not constant in many ErbB2-positive breast cancers. ErbB2 protein expression is heterogeneous (i.e. some parts of the tumor produce ErbB2 and some do not) in a significant fraction of ErbB2-positive breast cancers that display a relatively homogeneous amplification of the ErbB2 gene (the latter is normally detected by Fluorescence In Situ Hybridization (FISH)) [52]. Importantly, patients with heterogeneous ErbB2 expression in breast tumors have a worse prognosis than those with a homogeneous ErbB2 expression [51]. What factors control this heterogeneity is not known. Our data indicate that the activity of Mek or that of its effectors could be responsible for this phenomenon. Our results suggest that tumor cells with lower Mek activity produce lower ErbB2 amounts than those with a higher Mek activity.

Under what conditions could Mek activity be expected to be reduced in breast tumors? We found that an increase in the cell density within a three-dimensionally grown mass of breast cancer cells strongly blocks the expression and phosphorylation of the Mek substrate Erk and essentially eliminates ErbB2 from the cells (Figure 6A). (The mechanisms by which Erk expression is reduced under these conditions are presently not known and represent an important direction for our future studies). These data are consistent with observations that growth of tumors composed of ErbB2-positive breast cancer cells injected in immunodeficient mice is suppressed by trastuzumab much more efficiently if the drug is administered immediately after cell injection (before the tumor mass is formed) compared to the scenario when the drug is administered after a tumor mass is established [47]. Situations when a change in the breast tumor cell density could take place in breast cancer patients and thus affect ErbB2 levels have been described [46]. For example, it was found that circulating breast cancer cells (that are likely relatively sparse) are ErbB2-positive in a substantial number of cases while primary tumor masses (in which tumor cell density is likely higher than that in case of the circulating tumor cells) derived from the same patients are often ErbB2-negative [46]. Testing whether the circulating tumor cells display higher phospho-Erk levels than those composing respective primary ErbB2-negative tumors would represent a promising direction of the studies aimed at verifying the role of Mek/Erk activity in ErbB2 expression in breast cancer patients.

Primary tumors showing equivocal results of the immonohistochemical measurement of the ErbB2 levels are normally tested for ErbB2 gene amplification by FISH [53]. If the ErbB2 gene is amplified, respective patients receive trastuzumab-based therapies even though their tumors do not display high levels of ErbB2, and the indicated treatments can provide benefit to these patients [53]. The reasons why such therapies may be effective in such cases are unclear. Our study provides a potential mechanistic explanation of these observations. Respective primary tumors carrying an amplified ErbB2 gene might display low ErbB2 levels due to low Mek/Erk activity. However, cancer cells remaining in the body after the primary tumor is resected could have a higher Mek/Erk activity and ErbB2 expression than the cells in the primary tumor and thus be at least partially sensitive to subsequent trastuzumab-based treatments.

## MATERIALS AND METHODS

### Materials

The following compounds were used in this study. Selumetinib (Santa Cruz Biotechnology or ApexBio), Bafilomycin A1 (Sigma), trastuzumab (Roche), polybrene (Sigma-Aldrich).

### Cell culture

MCF-10A cells and their derivative MCF-ErbB2 were provided by M. Reiginato (Drexel University, USA). A variant of MCF10A cells MCF-MekDD producing and activated Mek2 mutant was generated as previously described [26, 28]. Expression vector encoding the activated Mek2 mutant was provided by M. Reiginato [26, 28]. The cells listed above were cultured in the medium containing DMEM/F12 (GIBCO) supplemented with 5% horse serum (GIBCO), 20 ng/ml EGF (Invitrogen), 10 μg/ml insulin (Novolin GE Toronto), 0.1 μg/ml cholera toxin (Biolynx), 0.5 mg/ml hydrocortisone (Sigma), 50 U/ml penicillin (GIBCO), 50 μg/ml streptomycin (GIBCO), 0.146 mg/ml L-glutamine (GIBCO). BT-474 (ATCC) were cultured in Hybri-Care medium (ATCC), 10% fetal bovine serum (Sigma), 100 U/ml penicillin (GIBCO), 100 μg/ml streptomycin (GIBCO), 0.29 mg/ml L-glutamine (GIBCO). AU-565 (ATCC) and HCC1419 (ATCC) were cultured in RPMI1640 medium (GIBCO), 10% fetal bovine serum (Sigma), 100 U/ml penicillin (GIBCO), 100 μg/ml streptomycin (GIBCO), 0.29 mg/ml L-glutamine (GIBCO). 293T cells (provided by Dr. A. Stadnyk (Dalhousie University) were cultured in DMEM (GIBCO), 10% fetal bovine serum (Sigma) 100 U/ml penicillin (GIBCO), 100 μg/ml streptomycin (GIBCO), 0.29 mg/ml L-glutamine (GIBCO). To detach cells from the ECM, they were plated in suspension culture above a layer of 1% sea plaque agarose polymerized in respective culture medium not containing any of the additional ingredients listed above.

### Western blot

This assay was performed as previously described [54]. The following antibodies were used: anti-ErbB2 (Cell Signaling Technology), anti-Erk (Cell Signaling Technology), anti-phospho-Erk (Cell Signaling Technology), anti-Mek (Cell Signaling Technology), anti-Atg12 (Cell Signaling Technology), anti-β-actin (Santa Cruz Biotechnology), anti-GAPDH (Cell Signaling Technology), anti-CDK4 (Santa Cruz Biotechnology).

### RNA interference

RNA interference was performed as we described [14]. The sequences of the sense strands of the RNAs used in this study were as follows: control RNA (siCONTROL non-targeting siRNA #1 (Dharmacon)), UGUUGUUUGAGGGGAACGGTT; human-specific ErbB2 siRNA 17, UGGAAGAGAUCACAGGUUA; human-specific ErbB2 siRNA 18 GAGACCC GCUGAACAAUAC. The latter two RNAs were from Dharmacon.

To knock down Atg 12 in BT474 cells 3×10^6^ 293T cells were cultured on a 100 mm tissue culture dish overnight and transfected with 3 μg of pLKO.1 non-targeting shRNA control expression vector encoding a control non-targeting RNA or pLKO.1-ATG12 shRNA 393 expression vector or pLKO.1-ATG12 shRNA 777 expression vector each encoding a separate Atg12-specific shRNA, referred to in this manuscript as Atg12 shRNA 1 and 2, respectively (the indicated 3 vectors were from Sigma-Aldrich) as well as 2.25 μg of psPAX2 and 0.75 μg of pMD2. G lentitroviral packaging vectors (both provided by L. Attardi, Stanford University, USA) in the presence of 12 μl of Lipofectamine 2000 (Invitrogen) in 6ml of OPTI MEM medium (GIBCO). The medium was changed 4 hours later to the regular cell growth medium, collected 48 hours later and filtered through a 0.45-micron filter unit. 0.5 ml of the viral supernatant and 8 μg/ml polybrene were then added to 3×10^5^ BT474 cells grown on a 60 mm tissue culture dissh, and the cells were cultured for 24 hours. The medium was then changed to the fresh medium and the cells were grown for 7 days in the presence of 2μg/ml puromycin.

### qPCR

This procedure was performed as we described [55]. The sequences of the primers used to amplify the ErbB2 mRNA were as follows: ACCTGCTGAACTGGTGTATG and TGACATGGTTGGGACTCTTG. The sequences of the primers used to amplify the 18S rRNA were as follows: ATAGTCAAGTTCGACCGTCTTC and GTTGATTAAGTCCCTGCCCTT.

### The following assays were performed as we described

Counting cells in suspension [36] as well as the soft agar growth assay [54].

### Statistical analysis

Statistical analysis of the data in Figure 6C and Supplementary Figures 1-5 was performed by the chi-square test for goodness-of-fit. Statistical analysis of all other data was performed by the Student's t-test.

### Western blot images

Western blot images were generated by Photoshop.

### Densitometry analysis of western blot images

This analysis was performed as we previously described [56].

## SUPPLEMENTARY MATERIALS FIGURES



## Abbreviations

ECM: extracellular matrix
EGFR: Epidermal Growth Factor Receptor
siRNA: small interfering RNA
shRNA: small hairpin RNA
qPCR: quantitative PCR
CHX: cyclohexamide
