# Supplementary material for: Mek activity is required for ErbB2 expression in breast cancer cells detached from the extracellular matrix
Source: Oncotarget. 2017 Oct 31;8(62):105383–96. doi: 10.18632/oncotarget.22194 (PMC5739645; doi:10.18632/oncotarget.22194)
Supplement: Supplementary file 1 [file oncotarget-08-105383-s001.pdf]

Mek activity is required for ErbB2 expression in breast cancer cells detached from the extracellular matrix

SUPPLEMENTARY MATERIALS

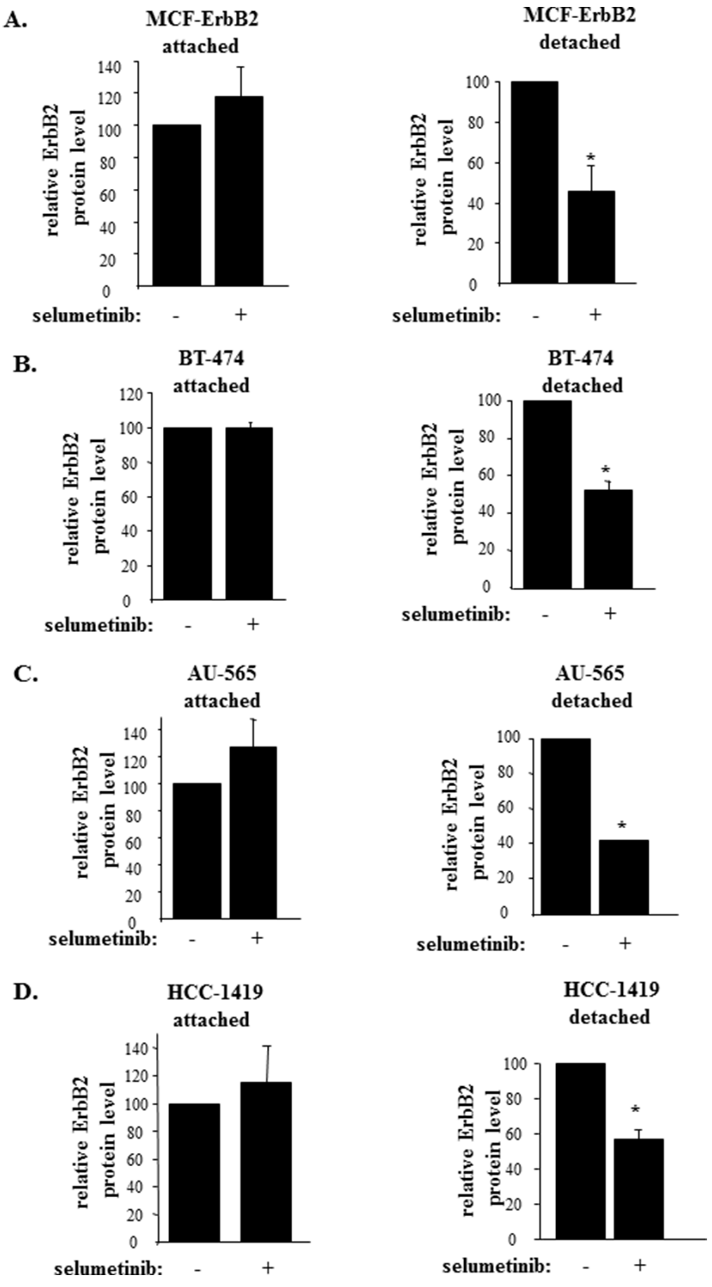

Supplementary Figure 1: Quantification of selumetinib-induced changes in ErbB2 protein levels in breast cancer cells. MCF-ErbB2 **(A)**, BT-474 **(B)**, AU-565 **(C)** and HCC-1419 cells **(D)** were cultured attached to (attached) or detached from (detached) the ECM in the presence of DMSO (-) or 1µM selumetinib (+) for 5h and assayed for ErbB2 expression by western blot. CDK4 was used as a loading control. Protein amounts were quantified by densitometry. ErbB2 levels were normalized by the levels of the loading control. ErbB2 levels in the control cells were designated as 100%. The data represent the average of two independent experiments plus SD. * indicates that p value was < 0.05.

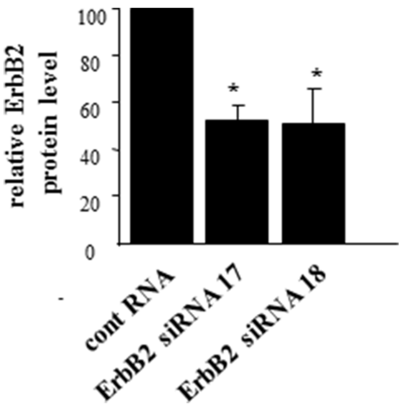

Supplementary Figure 2: Quantification of the changes in ErbB2 protein levels after ErbB2 knockdown in MCF-ErbB2 cells. MCF-ErbB2 cells were transfected with 25nM control RNA or 25 nM ErbB2-specific siRNA (ErbB siRNA) 17 or 18, cultured detached from the ECM for 24 and assayed for ErbB2 expression by western blot. β-actin was used as a loading control. Protein amounts were quantified by densitometry. ErbB2 levels were normalized by the levels of the loading control. ErbB2 levels in the cells transfected with a control RNA were designated as 100%. The data represent the average of two independent experiments plus SD. * indicates that p value was < 0.05.

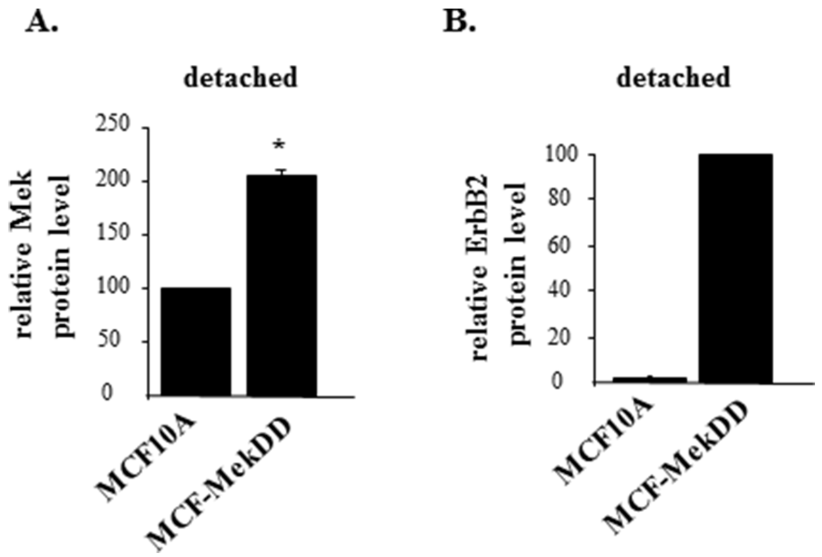

Supplementary Figure 3: Quantification of the changes in Mek and ErbB2 protein levels in MCF10A and MCF-ErbB2 cells. Indicated cells were cultured detached from the ECM for 3h and assayed for Mek **(A)** or ErbB2 **(B)** expression by western blot. β-actin was used as a loading control. Protein amounts were quantified by densitometry. Mek (A) and ErbB2 (B) levels were normalized by the levels of the loading control. Mek levels in MCF-10A cells (A) and ErbB2 levels in MCF-MekDD cells were designated as 100%. The data represent the average of two independent experiments plus SD. * indicates that p value was < 0.05.

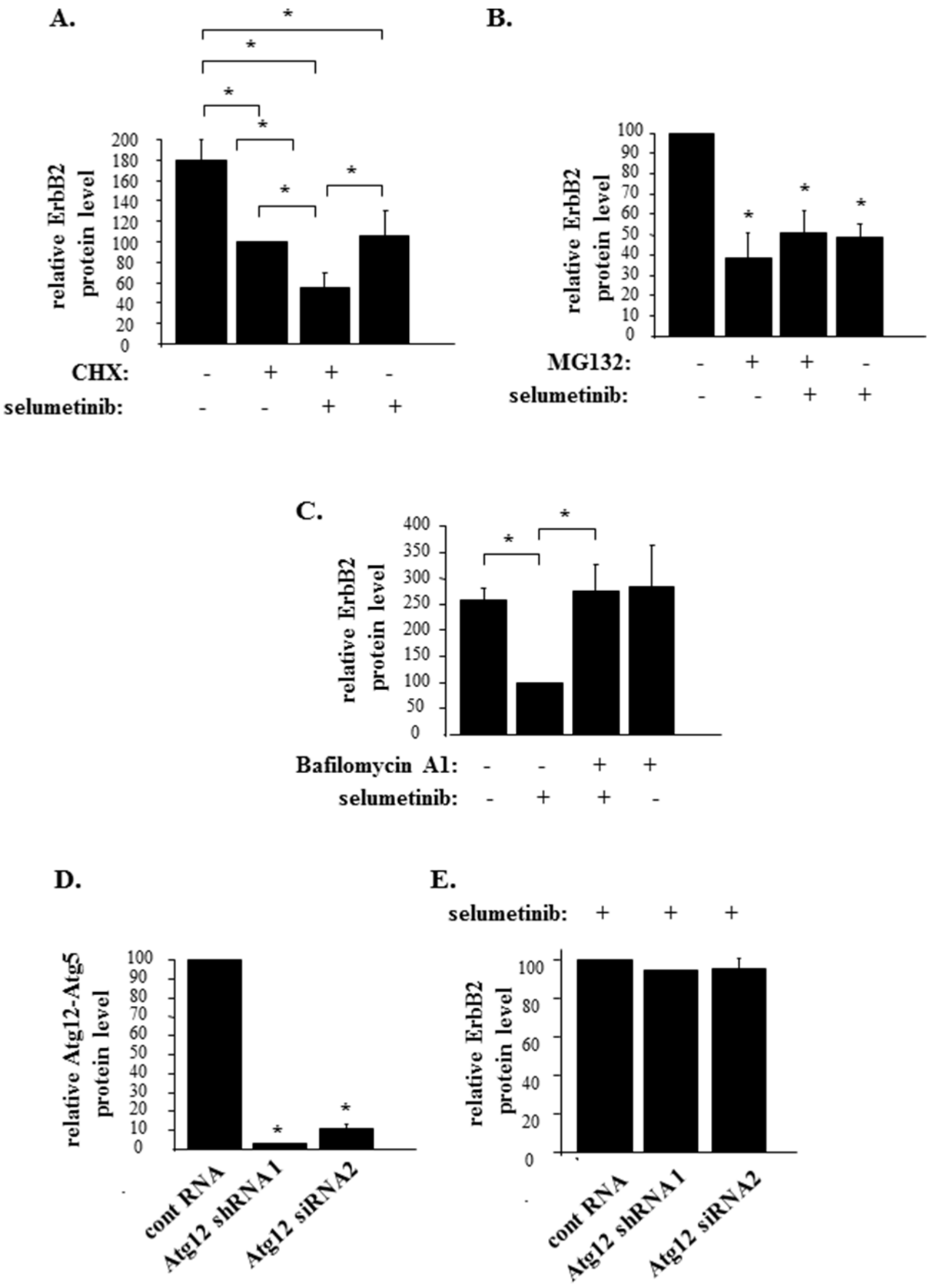

Supplementary Figure 4: Quantification of the changes in ErbB2 and Atg12 protein levels induced by selumetinib, MG132, Bafilomycin A1 and Atg12-specific shRNAs in breast cancer cells. (A). BT-474 cells were cultured detached from the ECM in the presence of DMSO (-) or 1μM selumetinib (+) or 10μg/ml cyclohexamide (CHX) (+) for 3h and assayed for ErbB2 expression by western blot. **(B)**. BT-474 cells were cultured detached from the ECM in the presence of DMSO (-) or 1μM selumetinib (+) or 10μg/ml MG132 (MG132) (+) for 5h and assayed for ErbB2 expression by western blot. **(C)**. BT-474 cells were cultured detached from the ECM in the presence of DMSO (-) or 1μM selumetinib (+) or 100 nM Bafilomycin A1 (+) for 5h and assayed for ErbB2 expression by western blot. **(D)** BT-474 cells were infected with retroviruses encoding the control RNA (cont RNA) or Atg12-specific shRNA 1 or 2 (Atg12 shRNA 1 or 2) and a puromycin resistance gene, the cells were expanded in the presence of puromycin and the resulting stable cell lines were assayed for Atg12 expression by western blot by use of the anti-Atg12 antibody. **(E)** Cells generated as in (D) were cultured detached from the ECM in the presence of 1μM selumetinib (+) for 5h and assayed for ErbB2 levels by western blot. GAPDH (A) and β-actin (B-E) was used as loading controls. Protein amounts were quantified by densitometry. Levels of ErbB2 (A-C, E) or Atg12-Atg5 complex (D) were normalized by the levels of the respective loading control. Protein levels observed in cyclohexamide- (A) or selumetinib- (C) treated cells or were designated as 100%. In all other cases protein levels observed in the control cells were designated as 100%. The data represent the average of two independent experiments plus SD. * indicates that p value was < 0.05.

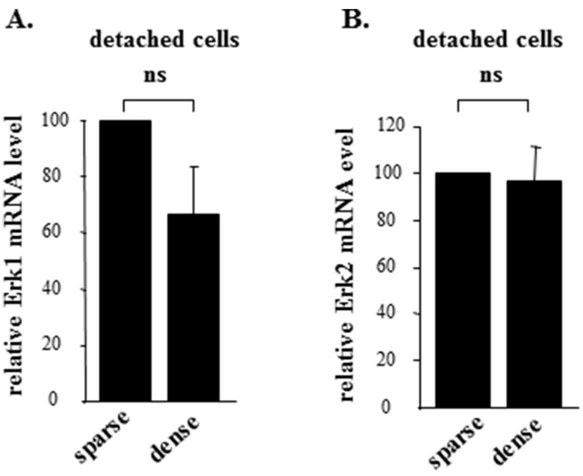

Supplementary Figure 5: Erk1 and Erk2 mRNA levels are not reduced by an increased density of detached breast tumor cells. (A, B) BT-474 cells were cultured detached from the ECM for 6 days at a concentration 12500 cells/ml (sparse) or 125000 cells/ml (dense) and assayed for Erk1 (A) and Erk 2 (B) mRNA levels by qPCR. The Erk1 or Erk2 mRNA levels were normalized by the levels of 18S rRNA which were also determined by qPCR. The resulting Erk1 or Erk2 mRNA levels in the sparse cells were designated as 100%. The data in (A, B) represent the average of three independent experiments plus SD. ns- not significant.

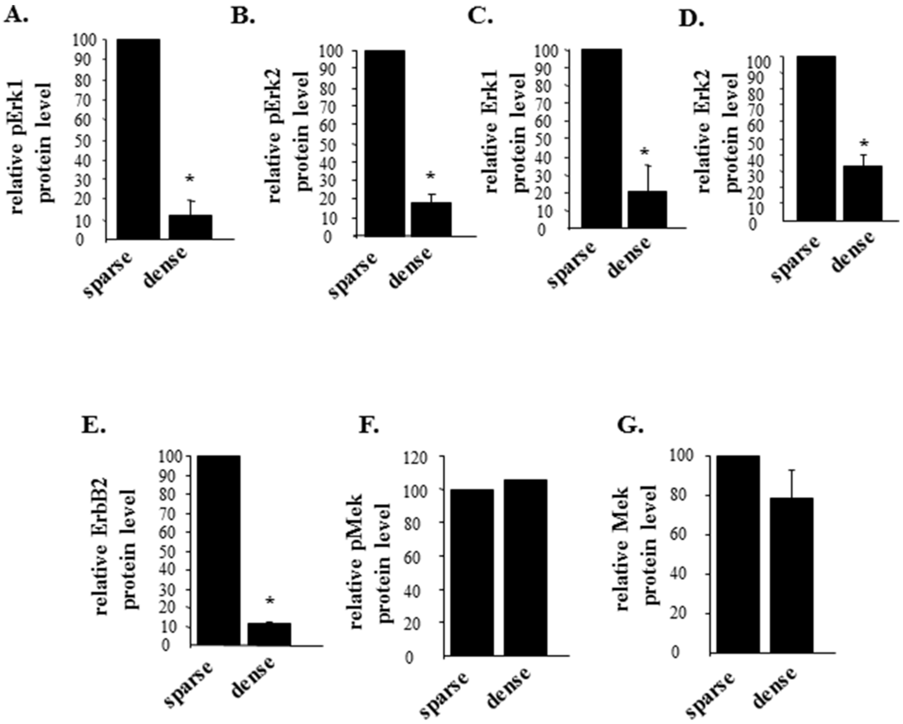

Supplementary Figure 6: Quantification of the changes in phospho-Erk, Erk, phospho-Mek, Mek and ErbB2 protein levels caused by an increased density of breast tumor cells. BT-474 cells were cultured detached from the ECM for 6 days at a concentration 12500 cells/ml (sparse) or 125000 cells/ml (dense) and assayed for phospho-Erk1 **(A)**, phospho-Erk2 **(B)**, Erk1 **(C)**, Erk2 **(D)**, ErbB2 **(E)**, phospho-Mek **(F)** and Mek **(G)** expression by western blot. β-actin was used as a loading control. Protein amounts were quantified by densitometry. Levels of the proteins indicated above were normalized by the levels of the loading control. The resulting protein levels in the sparse cells were designated as 100%. The data represent the average of two independent experiments plus SD. * indicates that p value was < 0.05.
